# Knowledge level of cancer symptoms and risk factors in the Gaza Strip: a cross-sectional study

**DOI:** 10.1186/s12889-020-08553-4

**Published:** 2020-03-30

**Authors:** Mohamedraed Elshami, Alaa Elshami, Nabeela Alshorbassi, Mohammed Alkhatib, Iyad Ismail, Khitam Abu-Nemer, Mustafa Hana, Ahmed Qandeel, Ahmed Abdelwahed, Hamza Yazji, Hisham Abuamro, Ghadeer Matar, Ahmed Alsahhar, Ahmed Abolamzi, Obay Baraka, Mahmood Elblbessy, Tahani Samra, Bettina Bottcher

**Affiliations:** 1 Ministry of Health, Gaza, Palestine; 2 Harvard Medical School, Boston, MA USA; 3 Faculty of Medicine, Islamic Unviersity of Gaza, Gaza, 108 Palestine

**Keywords:** Cancer awareness, Adolescent health, Cancer risk factors, Cancer signs and symptoms, Low- and middle-income countries, Early presentation, Gaza, Palestine

## Abstract

**Background:**

In low-income settings, cancer is often diagnosed in advanced stages due to late presentation. Good public awareness of cancer signs and symptoms has a positive impact on the time patients take before they present to healthcare professionals. Therefore, this study examined public knowledge of cancer signs and symptoms as well as risk factors in Gaza.

**Methods:**

This was a cross-sectional study. Participants were recruited from adult visitors (≥18 years) to governmental hospitals covering all five governorates of Gaza, and adolescent students (15 to 17 years) from 10 high schools in corresponding locations. An Arabic version of the Cancer Awareness Measure (CAM) was completed in a face-to-face interview. It described demographic data and knowledge of: cancer prevalence, age-related risk, signs and symptoms as well as risk factors both in recall and recognition questions.

**Results:**

Of 3033 participants invited, 2886 completed the CAM (response rate = 95.2%). Adult mean age ± standard deviation was 33.7 ± 11.7 years and that of adolescents was 16.3 ± 0.8 years. Half of the participants (*n* = 1457, 50.5%) were adolescent (781 females; 53.6%) and 1429 (49.5%) were adult (702 females; 49.1%). About two thirds (*n* = 1885) thought about cancer as unrelated to age. Only 196 participants (6.8%) identified colorectal cancer as the most common cancer among men. Awareness of cancer signs/symptoms was poor to fair, where ‘lump’ was most commonly recognized (*n* = 2227, 77.2%) and ‘change of bowel habit’ the least (*n* = 670, 23.2%). Only 217 participants (7.5%) had a good level of recognizing risk factors with ‘smoking’ being the most identified and ‘eating less than five portions of fruits and vegetables a day’ the least. There was a higher likelihood for adults to identify most cancer signs/symptoms and risk factors than adolescents, except for recalling ‘unexplained pain’, ‘persistent cough/hoarseness’, ‘non-healing ulcer’, ‘smoking’, and ‘eating less than five portions of fruits and vegetables a day’.

**Conclusion:**

Public awareness of cancer signs/symptoms and risk factors needs to improve to facilitate early presentation and diagnosis in Gaza. Combining the delivery of public campaigns with tailored education to population groups, including the youth, may increase their knowledge and maintain its impact.

## Background

Cancer is responsible for about 9.6 million deaths worldwide in 2018 with 70% of these occurring in low- and middle-income countries [1]. In 2016, the incidence rate of cancer was 89.0 per 100,000 general population per year and the mortality rate was 29.7 per 100,000 general population per year in the Gaza Strip [2]. In fact, cancer is the second leading cause of mortality, after cardiovascular diseases, representing 10.6% of the total reported deaths [3].

In Gaza, breast cancer (BC) is the most common cancer representing 20.5% of all cancer cases and 36.9% of cancers among females with an incidence rate of 18.6 per 100,000 general population per year in 2016 [2]. Colorectal cancer (CRC) is the second most common cancer, responsible for 12.6% of cancers, with an incidence rate of 11.5 per 100,000 male population per year [2]. In fact, CRC is the most common cancer among males representing 13.2% of their cancers and the second common cancer, after BC, among females representing 9.1% of their cancers [3]. The most common cause of cancer deaths in 2017 was lung cancer with 19.6% of all cancer-related deaths, followed by CRC with 12.7% and BC by 11.6% [4].

Poor awareness of cancer symptoms and risk factors resulting in delayed presentation as well as low availability of screening programs and limited access to healthcare services contribute to cancer-related deaths in low- and middle-income countries. All of these factors could play a major role in the high mortality rates [5–9]. Previous studies conducted in the Gaza Strip showed low awareness of symptoms as well as risk factors for both BC and CRC [10, 11]. Some educational initiatives were held by local universities and institutions. However, the effectiveness of these initiatives has not been measured to see their impact on people’s knowledge about cancer.

This study aimed to assess Gazans’ awareness of: (i) the age-related risk and prevalence of cancer; (ii) cancer signs and symptoms; (iii) cancer risk factors, and to evaluate the differences in awareness between population groups, such as men and women as well as adults and adolescents.

## Methods

### Study design and population

This was a cross-sectional study conducted in September and October, 2017. It assessed cancer awareness in the Gaza Strip and compared it between adolescents vs. adults and females vs. males.

As an assessment tool, the Cancer Awareness Measure (CAM) questionnaire, which is a validated standardized measurement for cancer awareness in the general population, was used [12]. The questionnaire consisted of four sections; the first described the demographic data. The second evaluated the knowledge of cancer prevalence in Gaza and its age-related risk. The third comprised open-ended (recall) questions while the fourth included closed (recognition) questions allowing comparison between these two question types (recall vs recognition). A 3-point scale was used to evaluate the knowledge of possible symptoms and warning signs of cancer. A 5-point Likert scale was used to assess the awareness of cancer risk factors.

The CAM was translated from English to Arabic by two healthcare professionals with experience in health survey design and proficiency in both languages. Next it was back-translated into English by another two bilingual clinical researchers with similar experiences. Before starting data collection, a pilot study was conducted with 119 respondents to test the clarity of the questions of the Arabic version of the CAM.

Assessment of internal consistency was carried out on the used scales comprising 16 items. Cronbach’s alpha showed the questionnaire to reach acceptable reliability, α = 0.71. Most items appeared to be worthy of retention, resulting in a decrease in the alpha if deleted. Although it has not been validated, a similar questionnaire was used in some previous studies conducted in Arabic-speaking countries [13–18].

### Sampling methods

Governmental hospitals are the main entry point for healthcare services in Gaza [10, 11]. Therefore, adults, aged 18 years or over, admitted to or visiting those hospitals, were the target population. There are 13 governmental hospitals in the Gaza Strip [3]. From these, the largest three hospitals, located in three separate geographical locations in the Gaza Strip, were chosen for recruitment of participants by stratified sampling. This sampling area covered most of Gaza’s population producing a geographically representative sample. Simple random sampling was used to choose visitors or patients (elective inpatients and outpatients) who were approached by data collectors in waiting rooms and hospital beds. Patients or visitors to oncology departments were excluded from the study. A total of 1483 adult participants was approached in the included hospitals.

In parallel to this, stratified sampling was used to identify 10 high schools (out of 147 high schools in Gaza [19]), that were located in the same areas as the study hospitals to achieve uniformity of areas. Simple random sampling was then utilized to select six classes in each of those 10 schools. All students in the chosen 60 classes were invited to participate (*n* = 1550). Data collectors asked adolescents who were willing to participate to have a face-to face interview to complete the CAM. High school students are studying health-related topics in their curriculum, which gives the opportunity to explore their awareness of cancer.

Data collectors were trained to recruit participants and facilitate the completion of the CAM. They were also trained to administer the questionnaire to illiterate participants.

### Statistical analysis

Descriptive statistics were used for demographic data and participants’ knowledge of cancer prevalence and its age-related risk. Two open-ended questions regarding cancer signs/symptoms and risk factors were used to assess the participants’ ability to recall a cancer symptom or risk factor without help from the interviewer.

The level of knowledge was determined by a scoring system that was used in previous studies in the United Kingdom and Malaysia [20, 21]. Each correctly recalled cancer sign/symptom or risk factor was given one point and incorrect answers were given a zero point. This resulted in a total score ranging from 0 to 8, which was categorized into three categories; poor knowledge (0 to 2), fair knowledge (3 to 5), and good knowledge (6 to 8).

To evaluate the participant’s recognition, 16 closed questions (eight for cancer signs/symptoms and eight for cancer risk factors) were asked. A 3-point scale (no, I do not know, yes) was used to test the recognition of cancer signs/symptoms. Each correct answer (yes) was given one point and incorrect answers (no or do not know) were given no point. The total score was then calculated and was categorized using the same aforementioned scoring system. A 5-point Likert scale (1 = strongly disagree, 5 = strongly agree) was used to assess the recognition of cancer risk factors. Every correct answer (agree or strongly agree) was given one point, while other answers (strongly disagree, disagree or not sure) had no point. The total score for recognizing cancer risk factors was obtained and categorized in the same previously mentioned manner.

The variables of interest were the knowledge level of cancer signs, symptoms and risk factors. The chi-square test was used to compare the awareness of each cancer sign/symptom and risk factor as well as the knowledge level between the two sub-populations. Multivariable logistic regression was used to test the association between gender and age-group with recalling cancer signs/symptoms and risk factors. It was also utilized to test their association with recognizing the symptoms. Ordinal regression was used to test the association of age-group and gender with recognizing risk factors. In addition, it was utilized to examine the association between the recall and recognition levels with gender and the age-group. Data were analyzed using Stata software version 15.0 (StataCorp, College Station, Texas, United States).

## Results

### Characteristics of participants

Of 3033 participants invited, 2886 completed the CAM (response rate = 95.2%). Adult mean age ± standard deviation (SD) was 33.7 ± 11.7 years and that of adolescents was 16.3 ± 0.8 years. Half of the participants (*n* = 1457, 50.5%) were adolescent (781 females; 53.6%) and 1429 (49.5%) were adult (702 females; 49.1%) (Table 1).

**Table 1:** Summary of characteristics of the participants

Characteristic	n (%)	Mean age (±SD)
Gender		
Male	1403 (48.6)	24.9 (±11.5)
Female	1483 (51.4)	24.9(±12.2)
Age-group		
Adolescent	1457 (50.5)	16.3 (±0.8)
Adult	1429 (49.5)	33.7 (±11.4)
Total	2886 (100.0)	24.9 (±11.9)

*n* number of participants tested, *SD* standard deviation

### Knowledge of cancer age-related risk and prevalence

A total of 1885 participants (65.3%) thought that cancer is unrelated to age and 112 (3.9%) did not know if there was a relation at all (Table 2). About 77.0% (*n* = 2220) identified correctly BC as the most common cancer among women in Gaza. On the other hand, only 196 participants (6.9%) answered with CRC as the most common cancer among men in Gaza. Adult and male participants had a higher likelihood than adolescent and female participants to recognize the age-related risk and to identify the most common cancers in women and men in Gaza (*p* < 0. 001).

**Table 2:** Summary of knowledge of cancer age-related risk and its prevalence in Gaza

Question	Total (*n* = 2886) n (%)	Adolescents (*n* = 1457) n (%)	Adults (*n* = 1429) n (%)	*p* value	Females (*n* = 1483) n (%)	Males (*n* = 1403) n (%)	*p* value
In the next year, who is most likely to develop cancer? Someone in their:							
20’s	260 (9.0)	104 (7.1)	156 (10.9)	< 0.001	132 (8.9)	128 (9.1)	< 0.001
30’s	175 (6.1)	54 (3.7)	121 (8.5)		88 (5.9)	87 (6.2)	
40’s	183 (6.3)	64 (4.4)	119 (8.3)		82 (5.5)	101 (7.2)	
50’s	115 (4.0)	39 (2.7)	76 (5.3)		49 (3.3)	66 (4.7)	
60’s	94 (3.3)	31 (2.1)	63 (4.4)		33 (2.2)	61 (4.3)	
70’s	27 (0.9)	9 (0.6)	18 (1.3)		10 (0.7)	17 (1.2)	
80’s	35 (1.2)	6 (0.4)	29 (2.0)		13 (0.9)	22 (1.6)	
Cancer is unrelated to age	1885 (65.3)	1047 (71.9)	838 (58.7)		1063 (71.7)	822 (58.6)	
I do not know	112 (3.9)	103 (7.1)	9 (0.6)		13 (0.9)	99 (7.1)	
What do you think is the most common cancer in women?							
Breast cancer	2220 (76.9)	981 (67.3)	1239 (86.7)	< 0.001	1116 (75.3)	1104 (78.7)	0.010
Uterine cancer	63 (2.2)	29 (2.0)	34 (2.4)		30 (2.0)	33 (2.4)	
Cervical cancer	23 (0.8)	15 (1.0)	8 (0.6)		9 (0.6)	14 (1.0)	
Leukemia	21 (0.7)	8 (0.5)	13 (0.9)		6 (0.4)	15 (1.1)	
Colon cancer	11 (0.4)	2 (0.1)	9 (0.6)		6 (0.4)	5 (0.4)	
Others	37 (1.3)	16 (1.2)	21 (1.5)		15 (0.9)	22 (1.4)	
I do not know	511 (17.7)	406 (27.9)	105 (7.3)		301 (20.3)	210 (15.0)	
What do you think is the most common cancer in men?							
Lung cancer	620 (21.5)	244 (16.7)	376 (26.3)	< 0.001	284 (19.2)	336 (23.9)	< 0.001
Prostate cancer	627 (21.7)	241 (16.5)	386 (27.0)		322 (21.7)	305 (21.7)	
Leukemia	245 (8.5)	110 (7.5)	135 (9.4)		108 (7.3)	137 (9.8)	
Brain cancer	90 (3.1)	50 (3.4)	40 (2.8)		49 (3.3)	41 (2.9)	
Colon cancer	196 (6.8)	33 (2.3)	163 (11.4)		80 (5.4)	116 (8.3)	
Liver cancer	74 (2.6)	48 (3.3)	26 (1.8)		34 (2.3)	40 (2.9)	
Testicular cancer	46 (1.6)	32 (2.2)	14 (1.0)		26 (1.8)	20 (1.4)	
Others	214 (7.4)	148 (10.3)	66 (4.7)		100 (6.7)	114 (8.1)	
I do not know	774 (26.8)	551 (37.8)	223 (15.6)		480 (32.3)	294 (21.0)	

*n* number of participants tested

### Awareness of cancer signs and symptoms

In general, awareness of cancer symptoms/warning signs and its risk factors was low when open-ended (recall) questions were used and higher with closed (recognition) questions. Unexplained lump/swelling was the most commonly recognized cancer symptom (*n* = 2227, 77.2%), while change of bowel habit was the least (*n* = 670, 23.2%) (Table 3).

**Table 3:** Summary of awareness scores for cancer symptoms and signs between adolescents vs adults and between females vs males

Symptom/sign	Recall							Recognition						
	Total (*n* = 2886) n (%)	Adolescents vs Adults			Females vs Males			Total (*n* = 2886) n (%)	Adolescents vs Adults			Females vs Males		
		Adolescents (*n* = 1457) n (%)	Adults (*n* = 1429) n (%)	*p* value	Females (*n* = 1483) n (%)	Males (*n* = 1403) n (%)	*p* value		Adolescents (*n* = 1457) n (%)	Adults (*n* = 1429) n (%)	*p* value	Females (*n* = 1483) n (%)	Males (*n* = 1403) n (%)	*p* value
Unexplained swelling/lump	1145 (39.7)	536 (36.8)	609 (42.6)	0.001	690 (46.5)	455 (32.4)	< 0.001	2227 (77.2)	1111 (76.3)	1116 (78.1)	0.24	1215 (81.9)	1012 (72.1)	< 0.001
Unexplained pain	541 (18.7)	310 (21.3	231 (16.2)	< 0.001	285 (19.2)	256 (18.2)	0.50	1154 (40.0)	629 (43.2)	525 (36.7)	< 0.001	622 (41.9)	532 (37.9)	0.027
Unexplained bleeding	189 (6.5)	79 (5.4)	110 (7.7)	0.013	118 (8.0)	71 (5.1)	0.002	1005 (34.8)	486 (33.4)	519 (36.3)	0.10	538 (36.3)	467 (33.3)	0.09
Persistent cough/ hoarseness	64 (2.2)	40 (2.7)	24 (1.7)	0.052	34 (2.3)	30 (2.1)	0.78	892 (30.9)	344 (23.6)	548 (38.3)	< 0.001	462 (31.2)	430 (30.6)	0.77
Persistent change in bowel habit	66 (2.3)	18 (1.2)	48 (3.4)	< 0.001	32 (2.2)	34 (2.4)	0.63	670 (23.2)	183 (12.6)	487 (34.1)	< 0.001	311 (21.0)	359 (25.6)	0.003
Persistent difficulty swallowing	35 (1.2)	14 (1.0)	21 (1.5)	0.21	18 (1.2)	17 (1.2)	0.99	944 (32.7)	398 (27.3)	546 (38.2)	< 0.001	497 (33.5)	447 (31.9)	0.34
Non-healing ulcer	34 (1.2)	29 (2.0)	5 (0.3)	< 0.001	22 (1.5)	12 (0.9)	0.12	1127 (39.1)	545 (37.4)	582 (40.7)	0.07	569 (38.4)	558 (39.8)	0.44
Unexplained weight loss	569 (19.7)	217 (14.9)	352 (24.6)	< 0.001	307 (20.7)	262 (18.7)	0.17	1977 (68.5)	974 (66.8)	1003 (70.2)	0.053	1027 (69.3)	950 (67.7)	0.37

*n* number of participants tested

Adults demonstrated higher awareness than adolescents in recognizing all cancer signs and symptoms except ‘unexplained pain’. This was also true after adjusting for gender (OR = 0.77, 95% CI: 0.66–0.89, *p* = 0.001) (Table 4). Only 347 participants (12.0%) had a good level of recognizing cancer signs and symptoms; 277 adults (19.4%) vs. 70 adolescents (4.8%) (Table 5). There was a significant association between the age group and the level of recognition (*p* < 0.001). The ordinal regression model showed that being adult increased the likelihood to have a higher recognition by 67.0% (OR = 1.67, 95% CI: 1.45–1.94, *p* < 0.001), which increased to 69.0% after the adjustment of gender (OR = 1.69, 95% CI: 1.46–1.96, *p* < 0.001). In addition, gender had a significant association with the level of recognition (*p* = 0.031). In fact, females had a higher likelihood by 20.0% to have better recognition (OR = 1.20, 95% CI: 1.04–1.38, *p* = 0.015), which went further up to 23.0% after adjusting for the age-group (OR = 1.23, 95% CI: 1.06–1.42, *p* = 0.005). Women were more likely to recognize all cancer signs and symptoms except for ‘persistent change in bowel habits’. This was also noticed after the adjustment for the age-group (OR = 0.80, 95% CI: 0.67–0.96, *p* = 0.016).

**Table 4:** The association of age group and gender with recalling and recognizing cancer symptoms

Symptom/sign	Recall (*n* = 2886)				Recognition (*n* = 2886)			
	Female gender		Being adult		Female gender		Being adult	
	^**a**^Adjusted OR (95% CI)	***p*** value	^**b**^Adjusted OR (95% CI)	***p*** value	^**a**^Adjusted OR (95% CI)	***p*** value	^**b**^Adjusted OR (95% CI)	***p*** value
Unexplained swelling/lump	1.84 (1.58–2.14)	< 0.001	1.32 (1.13–1.53)	< 0.001	1.76 (1.48–2.10)	< 0.001	1.14 (0.96–1.36)	0.14
Unexplained pain	1.05 (0.87–1.27)	0.61	0.71 (0.59–0.86)	< 0.001	1.17 (1.01–1.36)	0.040	0.77 (0.66–0.89)	0.001
Unexplained bleeding	1.65 (1.21–2.24)	0.001	1.49 (1.10–2.01)	0.009	1.15 (0.98–1.34)	0.08	1.15 (0.98–1.34)	0.08
Persistent cough/ hoarseness	1.05 (0.64–1.73)	0.85	0.61 (0.36–1.01)	0.06	1.06 (0.90–1.24)	0.49	2.02 (1.72–2.37)	< 0.001
Persistent change in bowel habit	0.93 (0.57–1.51)	0.76	2.77 (1.60–4.79)	< 0.001	0.80 (0.67–0.96)	0.016	3.57 (2.96–4.32)	< 0.001
Persistent difficulty swallowing	1.02 (0.52–1.99)	0.95	1.54 (0.78–3.04)	0.22	1.10 (0.94–1.29)	0.22	1.65 (1.41–1.93)	< 0.001
Non-healing ulcer	1.64 (0.81–3.34)	0.17	0.18 (0.07–0.46)	< 0.001	0.95 (0.82–1.10)	0.49	1.15 (0.99–1.33)	0.07
Unexplained weight loss	1.17 (0.97–1.42)	0.09	1.88 (1.56–2.27)	< 0.001	1.08 (0.92–1.27)	0.33	1.17 (1.01–1.37)	0.049

*n* number of participants tested, *OR* odds ratio, *CI* confidence interval

^a^Adjusted for age group, ^b^Adjusted for gender

**Table 5:** The level of knowledge of cancer symptoms and signs between adolescents vs adults and between females vs males

Knowledge level	Recall							Recognition						
	Total (***n*** = 2886) n (%)	Adolescents vs Adults			Females vs Males			Total (***n*** = 2886) n (%)	Adolescents vs Adults			Females vs Males		
		Adolescents (***n*** = 1457) n (%)	Adults (***n*** = 1429) n (%)	***p*** value	Females (***n*** = 1483) n (%)	Males (***n*** = 1403) n (%)	***p*** value		Adolescents (***n*** = 1457) n (%)	Adults (***n*** = 1429) n (%)	***p*** value	Females (***n*** = 1483) n (%)	Males (***n*** = 1403) n (%)	***p*** value
Poor	2729 (94.6)	1385 (95.1)	1344 (94.1)	0.23	1397 (94.2)	1332 (94.9)	0.38	845 (29.3)	449 (30.8)	396 (27.7)	< 0.001	402 (27.1)	443 (31.6)	0.031
Fair	157 (5.4)	72 (4.9)	85 (5.9)		86 (5.8)	71 (5.1)		1694 (58.7)	938 (64.4)	756 (52.9)		896 (60.4)	798 (56.9)	
Good	0	0	0		0	0		347 (12.0)	70 (4.8)	277 (19.4)		185 (12.5)	162 (11.5)	

*n* number of participants tested

### Awareness of cancer risk factors

Smoking was the most frequently recognized cancer risk factor (*n* = 2215, 76.7%) and eating less than five portions of fruit and vegetables a day was the least (*n* = 514, 17.8%) (Table 6). Adults were more likely to have recall and recognition of all cancer risk factors except for recalling ‘smoking’ and ‘eating less than five portions of fruit and vegetables a day’, where they were less likely than adolescents to recall them by 15.0% (OR = 0.85, 95% CI: 0.73–0.99, *p* = 0.041) and 14.0% (OR = 0.86, 95% CI: 0.74–0.99, *p* = 0.046) respectively, after the adjustment for gender (Table 7). Only 217 participants (7.5%) had a good level of recognizing cancer risk factors; 175 adults (12.2%) vs. 42 adolescents (2.8%) (Table 8). There was a significant association between the age-group and the level of recalling and recognizing cancer risk factors (*p* = 0.037 and < 0.001, respectively). After adjustment for gender, being adult increased the likelihood to have a higher recall and recognition than being adolescent by 1.54 times (OR = 1.54, 95% CI: 1.04–2.29, *p* = 0.032) and 2.15 times (OR = 2.15, 95% CI: 1.86–1.48, *p* < 0.001), respectively. However, there was no independent association of gender with the level of recall and/or recognition.

**Table 6:** Summary of awareness scores for cancer risk factors between adolescents vs adults and between females vs males

Risk factor	Recall							Recognition						
	Total (***n*** = 2886) n (%)	Adolescents vs Adults		Females vs Males				Total (***n*** = 2886) n (%)	Adolescents vs Adults		Females vs Males			
		Adolescents (***n*** = 1457) n (%)	Adults (***n*** = 1429) n (%)	***p*** value	Females (***n*** = 1483) n (%)	Males (***n*** = 1403) n (%)	***p*** value		Adolescents (***n*** = 1457) n (%)	Adults (***n*** = 1429) n (%)	***p*** value	Females (***n*** = 1483) n (%)	Males (***n*** = 1403) n (%)	***p*** value
Smoking	1027 (35.6)	542 (37.2)	485 (33.9)	0.07	469 (31.6)	558 (39.8)	< 0.001	2215 (76.7)	1102 (75.6)	1113 (77.9)	0.16	1200 (80.9)	1015 (72.3)	< 0.001
Passive smoking	75 (2.6)	29 (2.0)	46 (3.2)	0.046	44 (3.0)	31 (2.2)	0.24	1544 (53.5)	738 (50.7)	806 (56.4)	0.002	815 (55.0)	729 (52.0)	0.11
Eating less than 5 portions of fruit and vegetables a day	1086 (37.6)	578 (39.7)	508 (35.5)	0.023	647 (43.6)	439 (31.3)	< 0.001	514 (17.8)	202 (13.9)	312 (21.8)	< 0.001	270 (18.2)	244 (17.4)	0.59
Being overweight	93 (3.2)	45 (3.1)	48 (3.4)	0.75	58 (3.9)	35 (2.5)	0.035	1201 (41.6)	525 (36.0)	676 (47.3)	< 0.001	630 (42.5)	571 (40.7)	0.35
Getting sunburnt more than once as a child	38 (1.3)	14 (1.0)	24 (1.7)	0.10	17 (1.1)	21 (1.5)	0.42	536 (18.6)	200 (13.7)	336 (23.5)	< 0.001	278 (18.7)	258 (18.4)	0.81
Being over 70 years old	31 (1.1)	6 (0.4)	25 (1.7)	< 0.001	16 (1.1)	15 (1.1)	0.98	731 (25.3)	48 (17.0)	483 (33.8)	< 0.001	355 (23.9)	376 (26.8)	0.08
Having a close relative with cancer	212 (7.3)	76 (5.2)	136 (9.5)	< 0.001	124 (8.4)	88 (6.3)	0.032	980 (34.0)	383 (26.3)	597 (41.8)	< 0.001	527 (35.5)	453 (32.3)	0.07
Doing less than 30 min of moderate physical activity 5 times a week	37 (1.3)	16 (1.1)	21 (1.5)	0.41	18 (1.2)	19 (1.4)	0.74	722 (25.0)	355 (24.4)	367 (25.7)	0.42	349 (23.5)	373 (26.6)	0.06

*n* number of participants tested

**Table 7:** The association of age-group and gender with recalling and recognizing cancer risk factors

Risk factor	Recall (*n* = 2886)				Recognition (*n* = 2886)			
	Female gender		Being adult		Female gender		Being adult	
	^**a**^Adjusted OR (95% CI)	***p*** value	^**b**^Adjusted OR (95% CI)	***p*** value	^**a**^Adjusted OR (95% CI)	***p*** value	^**b**^Adjusted OR (95% CI)	***p*** value
Smoking	0.70 (0.60–0.81)	< 0.001	0.85 (0.73–0.99)	0.041	1.63 (1.37–1.94)	< 0.001	1.16 (0.98–1.38)	0.09
Passive smoking	1.38 (0.87–2.21)	0.17	1.66 (1.04–2.66)	0.035	1.14 (0.98–1.32)	0.08	1.27 (1.10–1.47)	0.002
Eating less than 5 portions of fruit and vegetables a day	1.69 (1.45–1.97)	< 0.001	0.86 (0.74–0.99)	0.046	1.08 (0.89–1.31)	0.41	1.74 (1.43–2.12)	< 0.001
Being overweight	1.60 (1.04–2.45)	0.031	1.11 (0.74–1.68)	0.61	1.10 (0.95–1.28)	0.21	1.60 (1.38–1.86)	< 0.001
Getting sunburnt more than once as a child	0.78 (0.41–1.49)	0.45	1.74 (0.90–3.38)	0.10	1.05 (0.87–1.28)	0.58	1.94 (1.60–2.35)	< 0.001
Being over 70 years old	1.07 (0.53–2.17)	0.86	4.32 (1.77–10.57)	0.001	0.89 (0.75–1.06)	0.18	2.48 (2.08–2.95)	< 0.001
Having a close relative with cancer	1.41 (1.06–1.87)	0.019	1.94 (1.45–2.60)	< 0.001	1.20 (1.02–1.40)	0.023	2.03 (1.74–2.38)	< 0.001
Doing less than 30 min of moderate physical activity 5 times a week	0.91 (0.47–1.74)	0.77	1.34 (0.69–2.58)	0.38	0.85 (0.72–1.01)	0.06	1.07 (0.90–1.26)	0.46

*n* number of participants tested, *OR* odds ratio, *CI* confidence interval

^a^Adjusted for age group, ^b^Adjusted for gender

**Table 8:** The level of knowledge of cancer risk factors between adolescents vs adults and between females vs males

Knowledge level	Recall							Recognition						
	Total (***n*** = 2886) n (%)	Adolescents vs Adults			Females vs Males			Total (***n*** = 2886) n (%)	Adolescents vs Adults			Females vs Males		
		Adolescents (***n*** = 1457) n (%)	Adults (***n*** = 1429) n (%)	***p*** value	Females (***n*** = 1483) n (%)	Males (***n*** = 1403) n (%)	***p*** value		Adolescents (***n*** = 1457) n (%)	Adults (***n*** = 1429) n (%)	***p*** value	Females (***n*** = 1483) n (%)	Males (***n*** = 1403) n (%)	***p*** value
Poor	2780 (96.3)	1414 (97.1)	1366 (95.6)	0.037	1420 (95.8)	1360 (96.9)	0.09	1254 (43.5)	747 (51.3)	507 (35.5)	< 0.001	626 (42.2)	628 (44.8)	0.26
Fair	106 (3.7)	43 (2.9)	63 (4.4)		63 (4.2)	43 (3.1)		1415 (49.0)	668 (45.9)	747 (52.3)		749 (50.5)	666 (47.5)	
Good	0	0	0		0	0		217 (7.5)	42 (2.8)	175 (12.2)		108 (7.3)	109 (7.7)	

*n* number of participants tested

## Discussion

Basic knowledge of cancer risk factors, signs and symptoms is essential for early detection as well as early presentation to medical services. This is especially important in low-resource settings as the Gaza Strip, where no systematic screening program exists, and early presentation with symptoms is the main pathway to early diagnosis and potential cure in cancer treatment. The knowledge level of cancer symptoms and risk factors in Gaza ranged from poor to fair. A minority of the participants showed good level of recognition of cancer signs, symptoms and risk factors. However, when asking open-ended questions to name cancer signs, symptoms or risk factors, all participants were very challenged and more than 95% in all groups showed poor recall. In general, adults displayed higher awareness than adolescents and females demonstrated better knowledge than males.

In concordance with other studies, knowledge of cancer risk factors as well as signs and symptoms improved with age [10, 11, 22–25]. This is not surprising as informal learning through experiences in life and exposure to public education programs increases with age. Kyle et al. found that 66.9% of British adolescents (vs 71.9% in this study) believed cancer was not related to age [26]. Recently, the Palestinian Ministry of Health reported an increase of cancer incidence rates from 73.6 per 100,000 general population in 2012 to 89.0 in 2016 [2]. This increase has been a focus of discussion among the Gaza population and might possibly have led to the understanding of increasing cancer cases in all age groups. Furthermore, causes for increasing rates are still under investigation, but have been linked in the general public to the repeated wars on Gaza, increased awareness of the public and presentation to healthcare professionals with symptoms as well as increased awareness of possible signs and symptoms among doctors.

Similar to this study, adolescents and adults in previous studies most frequently reported unexplained mass/lump as a cancer symptom [17, 23, 26, 27]. This could be due to perceiving having a mass as a concerning sign of something unusual. It might also be linked to the linguistic link of ‘tumour’, which represents a mass, to cancer. However, less than 53% of both adults and adolescents recognized other cancer symptoms, which is similar to participants in China, India, the United Kingdom (UK) and Oman [17, 24, 28, 29]. Recognition of cancer symptoms was found to be similar in Australia, Canada, Denmark, Norway, Sweden and the UK, when investigated by the International Cancer Benchmarking Partnership [27]. However, survival rates varied with the UK and Denmark lagging behind. Reasons for this were thought to be greater barriers experienced by participants, resulting in later first presentation to the doctor [26, 27]. This in turn would increase the ‘patient interval’, the time elapsing from the first symptom to presentation to a doctor, leading to more advanced stages at the time of diagnosis [9, 30, 31]. Therefore, reducing barriers for first presentation is as important as improving knowledge and awareness of cancer symptoms [9, 32–35]. Barriers can be emotional, like being worried about possible results, embarrassment or fear to see a doctor, service-related such as difficulty getting an appointment with a doctor, or practical like being too busy to see a doctor or difficulty with arranging transport [9–11]. However, negative beliefs also have a strong impact, such as the conviction that there is no cure for cancer or the treatment is worse than the actual disease [23, 32, 33, 35]. Such negative thoughts have been found to vary greatly, within countries in different areas as well as among different countries, which has been coined the ‘place effect’ [34, 36, 37]. This ‘place effect’ might have an especially strong influence in the Gaza Strip, which is a geographically relatively isolated area, suffering from a 13-year siege, restricting travel and information exchange [38]. Furthermore, cancer remains connected to poor outcomes locally. Such beliefs might be a further factor promoting late presentation. When people believe that the visit to the doctor will not improve their outlook or prognosis, they are more likely to delay or avoid such visits [32, 33, 35].

Adolescents were extremely poor in recognition of cancer risk factors with only 2.8% having a good level of recognition. However, adults also showed poor recognition with 12.4% demonstrating a good level of knowledge. The discrepancy between the age groups was found despite the fact that health-related content had been introduced in schools over the last decades, raising expectations that adolescents might at least be nearly as good as adults in recognition of risk factors. The school curriculum content might be reflected in the fact that more adolescents than adults knew that ‘smoking’ and ‘eating less than five portions of fruit and vegetables a day’ were risk factors for cancer, both of which are facts actually ‘taught’ at school. In most studies ‘smoking’ was the most commonly recognized risk factor [18, 23, 24, 29, 39–41]. Smoking enjoyed worldwide high publicity as a cancer risk factor and it is interesting that adolescents were better at recognizing this risk factor than adults. However, except for smoking as a risk factor, general awareness rates were poor in this study compared to other studies, which showed proportions of 60–88% recognizing smoking, 21–50% recognizing ‘eating less than five portions of fruit and vegetables a day’ and around 30% recognizing the importance of exercise, compared to 76.7, 17.8 and 25.0% respectively in this study [18, 22, 29, 41]. This discrepancy could be due to the fact that a number of awareness campaigns have been done on factors such as smoking, exercise and healthy diet in the localities of these other studies [27, 40, 42–44]. However, so far, no sustained public awareness campaigns on the potential negative impact of modifiable lifestyle factors on cancer have been evaluated as to their impact on public knowledge or awareness in the Gaza Strip. Although around half of all cancer in the U.S. have been attributed to modifiable factors, such as smoking, lack of exercise and unhealthy diets by the American Cancer Society [45], this has not been translated into major interventions in low-income settings, such as Gaza. Therefore, an urgent need exists to include low- and middle-income countries in such efforts.

### Strengths and limitations

This study took a large and representative sample from the Gaza population, including all five governorates. Moreover, it included adolescents from government schools, opening an unprecedented view on their awareness and knowledge around cancer. However, as this study aimed at assessing knowledge and awareness of cancer, it could not directly link awareness levels to actual outcomes. For this, another study design will be needed. In addition, adult participants were recruited from among hospital visitors which might have caused a degree of selection bias, as these people might have displayed a greater degree of health-seeking behaviour, possibly based on greater baseline health awareness. Furthermore, the paucity of sociodemographic data, such as level of education, which could influence the cancer awareness level, made analysis and examination of other factors influencing cancer awareness very challenging.

### Implications for practice

For greater impact, raising awareness has to be combined with careful promotion or reinforcement of positive beliefs and information of possible chances of cure [33, 35]. Raising such awareness among adolescents could be a useful future investment and give an opportunity for early preventive measures. Kyle et al. showed that a school-based educational intervention resulted in improving the recall and recognition of most of the cancer signs and symptoms even after 6 months from the intervention [26]. Such an intervention might be especially effective when combined with addressing negative beliefs. By reducing negative beliefs and increasing awareness in the younger age-group, a sustained effect on reduction of late presentation might be achieved, which could have a pronounced effect in low-resource settings, such as the Gaza Strip, in improving the quality of life and increasing survival of cancer sufferers. Currently, survival rates are poor in Palestine with 60% of deaths due to breast cancer in 2016 (643 of 1072 deaths) having been judged as ‘prematurely’ [46]. Such poor survival has its main reasons in the lack of systematic and organized screening programs and late presentation to healthcare professionals. Therefore, educational intervention in younger age groups could make a fundamental difference to survival and quality of life in cancer patients in the Gaza Strip. Furthermore, interventions to improve public awareness of cancer symptoms have been shown to be more effective when delivered to individuals rather than with a population-based approach, such as in public awareness campaigns [25, 47–50]. Therefore, in low-resource settings, lacking systematic and well-organized screening programs, where early diagnosis is essential to improve survival, a population-based approach should be combined with more tailored individualized education for better results [32, 35, 43, 47]. The effects from such interventions could be pronounced and sustainable, when involving younger age groups, such as adolescents [24], as reflected in this study, by the better knowledge demonstrated by adolescents of some risk factors, included in their health-related school curriculum.

## Conclusions

Both adults and adolescents in this low-income setting, the Gaza Strip, showed a big knowledge gap in the recall and recognition of cancer symptoms and risk factors, when compared to studies from other high-income settings. However, information delivered in the school curriculum might have made an impact on adolescents’ health-related knowledge. Therefore, combining the delivery of public campaigns as well as tailoring knowledge to population groups, including the youth, may increase the impact and sustainability to meet this urgent need in low- and middle-income areas, including the Gaza Strip.

## Data Availability

The dataset used and analyzed during the current study is available from the corresponding author on reasonable request.

## Abbreviations

BC: Breast cancer
CRC: Colorectal cancer
CAM: Cancer awareness measure
CI: 95% confidence interval
OR: Odds ratio
UK: United Kingdom
US: United States
